# Isovolumic loading of the failing heart by intraventricular placement of a spring expander attenuates cardiac atrophy after heterotopic heart transplantation

**DOI:** 10.1042/BSR20180371

**Published:** 2018-06-27

**Authors:** Martin Pokorný, Iveta Mrázová, Jan Šochman, Vojtěch Melenovský, Jiří Malý, Jan Pirk, Lenka Červenková, Janusz Sadowski, Zdeněk Čermák, Karel Volenec, Šárka Vacková, Hana Maxová, Luděk Červenka, Ivan Netuka

**Affiliations:** 1 Department of Cardiovascular Surgery, Institute for Clinical and Experimental Medicine, Prague, Czech Republic; 2 Department of Pathophysiology, Second Faculty of Medicine, Charles University, Prague, Czech Republic; 3 Center for Experimental Medicine, Institute for Clinical and Experimental Medicine, Prague, Czech Republic; 4 Department of Cardiology, Institute for Clinical and Experimental Medicine, Prague, Czech Republic; 5 Department of Renal and Body Fluid Physiology, Mossakowski Medical Research Centre, Polish Academy of Sciences, Warsaw, Poland; 6 ELLA-CS, Ltd., Hradec Králové, Czech Republic

**Keywords:** Cardiac atrophy, heterotopic heart transplantation, heart failure, mechanical heart unloading, spring expander

## Abstract

Cardiac atrophy is the most common complication of prolonged application of the left ventricle (LV) assist device (LVAD) in patients with advanced heart failure (HF). Our aim was to evaluate the course of unloading-induced cardiac atrophy in rats with failing hearts, and to examine if increased isovolumic loading obtained by intraventricular implantation of an especially designed spring expander would attenuate this process. Heterotopic abdominal heart transplantation (HT_x_) was used as a rat model of heart unloading. HF was induced by volume overload achieved by creation of the aorto-caval fistula (ACF). The degree of cardiac atrophy was assessed as the weight ratio of the heterotopically transplanted heart (HW) to the control heart. Isovolumic loading was increased by intraventricular implantation of a stainless steel three-branch spring expander. The course of cardiac atrophy was evaluated on days 7, 14, 21, and 28 after HT_x_. Seven days unloading by HT_x_ in failing hearts sufficed to substantially decrease the HW (−59 ± 3%), the decrease progressed when measured on days 14, 21, and 28 after HT_x_. Implantation of the spring expander significantly reduced the decreases in whole HW at all the time points (−39 ± 3 compared with −59 ± 3, −52 ± 2 compared with −69 ± 3, −51 ± 2 compared with –71 ± 2, and −44 ± 2 compared with −71 ± 3%, respectively; *P*<0.05 in each case). We conclude that the enhanced isovolumic heart loading obtained by implantation of the spring expander attenuates the development of unloading-induced cardiac atrophy in the failing rat heart.

## Introduction

Heart failure (HF) represents a serious public health problem and, unless a real progress in HF prevention and treatment ensues, over the coming decade the number of new HF patients would be increasing yearly by 50% [1–3]. In patients with HF, the left ventricle (LV) undergoes complex adverse molecular, cellular, and gross structural changes, with increasing cardiomyocyte size and LV dimension (‘LV remodeling’), leading ultimately to further impairment of LV function and progression of HF [1]. Until recently, this remodeling process was believed to inevitably progress and the notion that HF represents the final consequence of irreversible heart damage was widely accepted [1–3]. However, studies on patients with implanted LV assist device (LVAD) which is applied at the end-stage HF have shown that such LV unloading largely prevented the cellular, molecular, electrophysiological, and structural abnormalities in the failing myocardium. This process is referred to as ‘reverse remodeling’ [3–8] and implantation of LVAD in patients with advanced HF has been introduced as a ‘bridge to recovery’. It was claimed that the improvement in the myocardial function observed after LVAD-induced mechanical unloading of the myocardium can eventually lead to a successful weaning from LVAD treatment, and sustained myocardial recovery can be achieved without the need for another LVAD implantation or heart transplantation (HT_x_) [3,6–9]. However, even though the signs of reverse remodeling are present in most patients after LVAD implantation, the recovery of myocardial function is rare. Apparently, for unclear reasons beneficial biological signs of reverse remodeling are not readily translated into clinical improvement [3–10].

Unfortunately, prolonged LVAD support was reported to induce cardiac atrophy, probably the main harmful effect in such treated patients. Most probably, cardiac atrophy underlies the lack of significant myocardial function improvement in LVAD patients, even though their hearts exhibit molecular, functional, and structural signs of reverse remodeling [4,6,11–15]. None of the approaches aimed at minimizing this detrimental effect proved successful. However, the major limitation of these attempts is that they were performed with unloaded normal (i.e. non-failing) hearts. In the absence of studies performed with failing hearts, the process of cardiac atrophy and the potential anti-atrophic mechanisms would remain virtually unknown [11,14–30]. Recent analyses of experimental studies of the process of unloading-induced cardiac atrophy pointed to the merits of implanting into the LV a device that would provide sufficient isovolumic loading [9,30]. However, the approach used previously, involving placement in the LV of an inflated balloon, cannot be applied in patients treated with LVAD [23]. Therefore, we developed a stainless steel three-branch spring expander which provides sufficient isovolumic loading and should not impair ejection function of the LV.

Therefore, the *first aim* of our present study was to examine if, in the failing heart, isovolumic loading obtained using this expander would attenuate the process of unloading-induced cardiac atrophy which normally follows heterotopic HT_x_ on the abdominal aorta of an isogenic rat recipient (an established model for studying effects of mechanical heart unloading).

Since it is believed that in the failing heart the return to the ‘*fetal cardiac genes*’ (cardiac gene expression of contractile proteins and of the genes controlling substrate uptake and substrate oxidation) is detrimental and contributes to the development of unloading-induced cardiac atrophy [30–32], the *second aim* of our study was to elucidate the effects of isovolumic loading on the expression of the ‘fetal cardiac gene program’.

## Methods

### Ethical approval, animals, and HT_x_ and HF models

The studies were performed in accordance with guidelines and practices established by the *Animal Care and Use Committee of the Institute for Clinical and Experimental Medicine*, Prague, and of the Second Faculty of Medicine, Charles University, Prague, in accord with the *European Convention on Animal Protection and Guidelines on Research Animal Use* and was approved by this committee and consequently by Ministry of Health of Czech Republic (the decision number for this project was 8421/2015-OZV-30-20.2.15 issued by Ministry of Health of Czech Republic)*.* Adult male Lewis rats (Charles River Laboratories, Velaz, Prague, Czech Republic), 10–11 weeks of initial age, and 340–360 g body weight, were used. The classical heterotopic heart transplantation (HT_x_) originally described by Ono and Lindsey [34] and employed and validated by many investigators was used as the model to simulate the effect of full mechanical unloading of the heart [14,16–30,35]. HF was induced by volume overload induced by aorto-caval fistula (ACF) created using needle technique as originally described by Garcia and Diebold [36] and then employed and validated by many investigators including our own group [37–40]. Ten weeks after ACF creation the animals were used as heart donors. Earlier studies, including ours, demonstrated that at that time ACF animals are in the stage of advanced, but still compensated HF but, when untreated, soon progress toward decompensated hypertrophy and HF [37,38,40].

### Experimental design

#### Effects of enhanced isovolumic loading induced by implantation of the spring expander into LV on the cardiac atrophy after heterotopic HT_x_ in failing hearts

**Figure 1:**
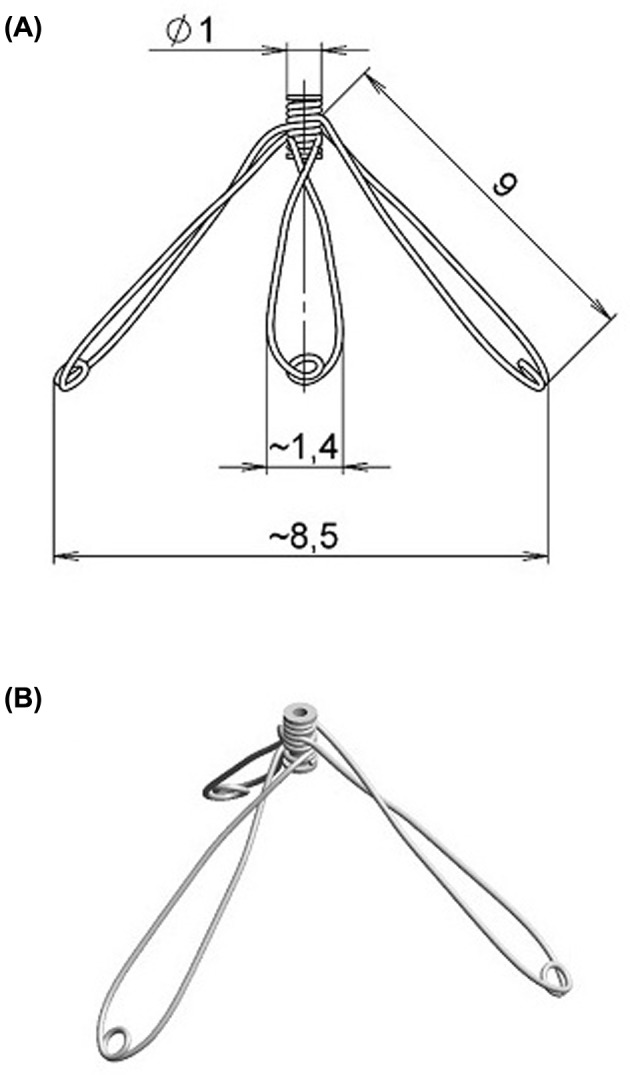
Diagramatic presentation of the spring expander Diagramatic presentation of the spring expander with branch lengths of 9 mm (**A**) and the general view of the device (**B**).

HT_x_ of failing heart was performed and, in appropriate groups, implantation into the LV of the stainless steel three-branch expander (briefly: ‘expander’) was performed through LV apex incision. Based on preliminary studies, for failing hearts the expanders with branch length of 9 mm were used (Figure 1). Notably, our preliminary acute echocardiographic studies followed by anatomic examination indicated that the implantation did not acutely impair ejection function of the LV in healthy as well as in failing hearts. The composition of the stainless wire (0.17 mm in diameter, 316 LVM, Fort Wayne Metal) used for expander construction, was (%): carbon 0.023, manganese 1.84, silicon 0.37, phosphorus 0.014, sulphur 0.001, chromium 17.57, nickel 14.68, molybdenum 2.79, copper 0.03, nitrogen 0.03, and iron to balance of 100%. Elastic and mechanical properties of both spring expanders were measured *in vitro* on the miniaturized compression device and analyzed by generation of stress–strain relationship as described by Lossef et al. [41]. The degree of cardiac atrophy was evaluated as the weight of total heart and, separately, of its individual structural components (i.e. LV + septum and right ventricle (RV)). Explicitly, an index of cardiac atrophy was calculated as the weight ratio of the heterotopically transplanted to the control heart, i.e. to the heart from animals with ACF-induced HF, examined 11, 12, 13, or 14 weeks after creation of ACF (experimental groups #9–12 in the list below). This was done so because only healthy Lewis rats can be used as recipients: the animals 11–14 weeks after creation of ACF develop decompensated HF [38,40] and would not survive the surgical procedure (as tested in our preliminary studies). The degree of cardiac atrophy was expressed as percent decreases in the whole heart weight (HW), LV and RV weights (RVW) of the hearts after HT_x_ compared with control. Unfortunately, for evaluation of the degree of cardiac atrophy we could not use HW of the donor’s heart before and after HT_x_. This was so because with the classical heterotopic HT_x_ the donor’s heart is immediately placed in cold cardioplegia solution, which precludes precise determination of HW [34,35]. The degree of cardiac atrophy was evaluated on days 7, 14, 21, and 28 after heterotopic HT_x_. The following groups (*n*=8 in each) were examined:

1. Lewis rats (recipient) + HT_x_ of failing donor’s heart (from ACF rats) (7 days after HT_x_),
2. Lewis rats + HT_x_ of failing heart (14 days),
3. Lewis rats + HT_x_ of failing heart (21 days),
4. Lewis rats + HT_x_ of failing heart (28 days),
5. Lewis rats + HT_x_ of failing heart + implantation of expander (7 days),
6. Lewis rats + HT_x_ of failing heart + implantation of expander (14 days),
7. Lewis rats + HT_x_ of failing heart + implantation of expander (21 days),
8. Lewis rats + HT_x_ of failing heart + implantation of expander (28 days),
9. ACF Lewis rats 11 weeks after creation of ACF
10. ACF Lewis rats 12 weeks after creation of ACF
11. ACF Lewis rats 13 weeks after creation of ACF
12. ACF Lewis rats 14 weeks after creation of ACF

**Figure 2:**
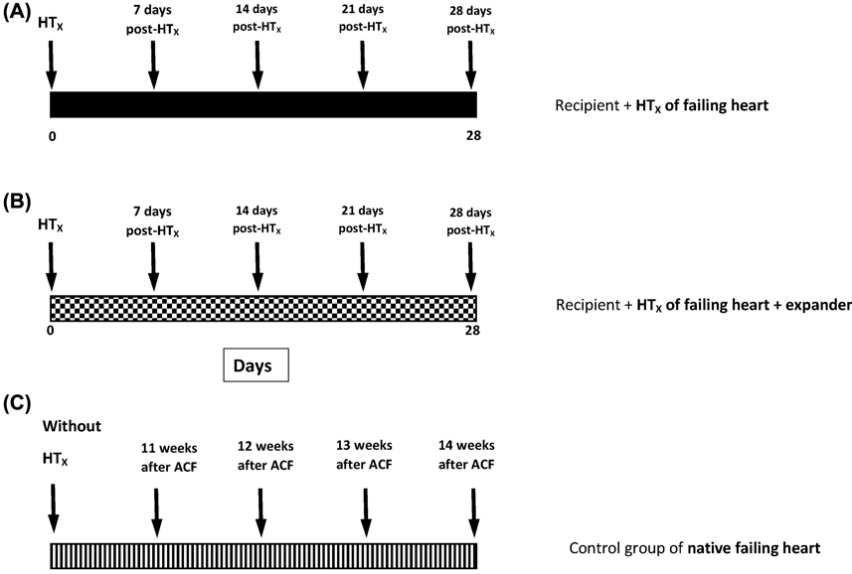
Experimental protocol An outline of the set of experimental groups: animals with HF elicited by creation of ACF after HT_x_ either without (**A**) or with (**B**) implantation of the expander, and the control groups of ACF animals without HT_x_ (**C**).

The experimental design used is outlined in Figure 2.

At the end of experiments, the hearts were excised, blood was removed from the chambers by gentle compression, and the hearts’ wet weight was determined.

In separate appropriately matched experimental groups (*n*=8 in each) the hearts were harvested for histological examination of the myocardium as described previously [39,42]. Briefly, rats were anesthetized with a combination of midazolam 5 mg.kg^−1^ (Dormicum, Roche Ltd., Prague, Czech Republic) and ketamine 50 mg.kg^−1^ i.p. (Calypsol, Gedeon Richter Ltd., Budapest, Hungary). Beating organs, i.e. the native heart (from the chest of ACF rats at an appropriate week) and the heart after HT_x_ (from the abdomen), with and without implanted expander, were perfused *in situ* with 20 ml Thomas cardioplegia solution and subsequently fixed in 4% paraformaldehyde in PBS and embedded into Tissue-Tek. The blocks were cut using a cryomicrotome, and cardiomyocyte width was measured in the subendocardium, midmyocardium, and subepicardium of the LV. The cardiomyocyte length was measured only in the midmyocardium. In each layer, 50 cardiomyocytes were assessed. To avoid underestimation, only the cells in which the nucleus was visible were measured. Since there were no significant differences in the cardiomyocyte width between the layers, the data from the subendocardium, midmyocardium, and subepicardium were pooled [39]. Analysis of LV and RV fibrosis was performed in sections stained with Picrosirius Red (Direct Red 80, Sigma–Aldrich, MO, U.S.A.) as described in detail previously [42,43]. Briefly, the interstitial collagen was analyzed in polarized light using ten images of the LV and five images of a RV scanned from a midmyocardium, without perivascular areas (magnification: 200×, microscope Nikon eclipse Ni-E, camera Nikon DS-L3, Tokyo, Japan). The percent area of myocardial fibrosis was calculated semiquantitatively, using the imaging software NIS-Elements Ar (LIM, Prague, Czech Republic). The measurements of the cardiomyocyte width and length, and of the degree of myocardial fibrosis were performed 7, 14, 21, and 28 days after heterotopic HT_x_. Histological examination had to be performed in separate groups of animals, because perfusion with cardioplegia solution with subsequent immediate fixation in paraformaldehyde solution precludes precise determination of the whole HW, and the LV or RV weights.

In addition, samples of LV myocardium were frozen in liquid nitrogen and stored at −80°C until analysis of the ‘fetal cardiac gene’ expressions, as described in detail previously. This was performed on days 7 and 28 after heterotopic HT_x_ [44]. Briefly, the relative gene expression was calculated by the ΔΔ*C*_t_ method and results were expressed as the n-fold difference in gene expression relative to *β-actin* mRNA of the transplanted-to-control heart (i.e. the native heart, normal, or the one from animals with HF at an appropriate week); this was done as described in our previous studies [43,45]. We measured isoform-specific transcription of myosin heavy chain (MHC), specifically, expressions of ‘adult’ isoform α (αMHC) and ‘fetal’ isoform β (βMHC), and sarcoendoplasmatic Ca^2+^-ATPase pumps (SERCA) as the paradigm of genes controlling ‘cardiac contractility efficiency’; expressions of glucose transporters type 1 (GLUT1), type 4 (GLUT4), and carnitine palmitoyltransferase I (CPT I), as the paradigm of genes controlling ‘cardiac substrate uptake and substrate oxidation’; expressions of atrial natriuretic peptide (ANP), transforming growth factor β1 (TGFβ1) and fibroblast growth factor type 2 (FGF-2) as paradigm genes generally reflecting pathological remodeling of the heart [31–33,46].

The primers were designed by Primer3 software and purchased from Generi Biotech Ltd. (Hradec Králové, Czech Republic). Primer sequences are collected in Table 1.

**Table 1:** Gene-specific primer sequences used for quantitative real-time PCR used in the present study

	Forward primer 5′–3′	Reverse primer 5′–3′
αMHC	AGTCAGAGAAGGAGCGCCTA	GGACACGATCTTGGCCTTGA
βMHC	CTGGAGCAGCAAGTGGATGA	GTCAGCTTCAGGTCACCCTC
SERCA	TTGTGGCCCGAAACTACCTG	GGGCTGGAAGATGTGTTGCT
GLUT1	CTGTAGGGCTGGACCTTTGG	AATGGAGCCTGGACCCCTAT
GLUT4	TACCGTCTTCACGTTGGTCTC	TAACTCATGGATGGAACCCGC
CPT I	GGACAGCAGGCACATTGTTG	TGGCTCTGAGGGATCATCCA
ANP	TGGAGGAGAAGATGCCGGTA	CTGAGACGGGTTGACTTCCC
TGFβ1	CTTTGTACAACAGCACCCGC	TAGATTGCGTTGTTGCGGTC
FGF-2	CGCACCCTATCCCTTCACAG	GCCTTCCACCCAAAGCAGTA

### Statistical analyses

All values are expressed as mean ± S.D. Using the GraphPad Prism software (GraphPad Software, San Diego, CA, U.S.A.), statistical analysis was performed by Student’s *t*test, Wilcoxon’s signed-rank test for unpaired data, or one-way ANOVA followed by Student–Newman–Keuls test when appropriate. Student’s *t*test was used for comparing differences in animals at the same time point. ANOVA was employed for evaluation of the differences within the same experimental group over time (i.e. changes on days 7, 14, 21, and 28 after HT_x_). Values exceeding the 95% probability limits (*P*<0.05) were considered statistically significant.

## Results

Table 2 summarizes HW, LV weights (LVW), and RVW of transplanted hearts in absolute values measured 7, 14, 21, and 28 days after HT_x_. Table 3 summarizes the same parameters for ACF animals that served as control values (100%) for evaluation of the process of cardiac atrophy of failing hearts 7, 14, 21, and 28 days after HT_x_.

**Table 2:** Body weight and weights of the transplanted heart (i.e. donor’s heart) and its individual structural components after HT_x_ (*n*=8 in each group)

	Parameter			
	BW	HW	LVW	RVW
	(g)	(mg)	(mg)	(mg)
Group				
Lewis rats (recipient) + HT_x_ of failing donor’s heart (from ACF rats) without expander (7 days after HT_x_)	397 ± 11	942 ± 48	657 ± 28	257 ± 19
Lewis rats + HT_x_ of failing donor’s heart without expander (14 days after HT_x_)	405 ± 9	801 ± 31	447 ± 21	195 ± 27
Lewis rats + HT_x_ of failing donor’s heart without expander (21 days after HT_x_)	414 ± 8	774 ± 29	445 ± 26	159 ± 19
Lewis rats + HT_x_ of failing donor’s heart without expander (28 days after HT_x_)	417 ± 9	764 ± 27	435 ± 27	157 ± 24
Lewis rats + HT_x_ of failing donor’s heart + implantation of expander (7 days after HT_x_)	400 ± 9	1401 ± 44*	806 ± 27*	284 ± 36*
Lewis rats + HT_x_ of failing donor’s heart + implantation of expander (14 days after HT_x_)	409 ± 9	1207 ± 37*	780 ± 29*	173 ± 27*
Lewis rats + HT_x_ of failing donor’s heart + implantation of expander (21 days after HT_x_)	418 ± 11	1306 ± 31*	825 ± 31*	191 ± 26*
Lewis rats + HT_x_ of failing donor’s heart + implantation of expander (28 days after HT_x_)	420 ± 13	1505 ± 37*	869 ± 37*	211 ± 31*

Values are means ± S.E.M. Abbreviation: BW, body weight. *P<0.05 compared with values from transplanted heart without implanted expander at the same time point.

**Table 3:** Body weight and weights of the native failing heart and its individual structural components after induction of ACF

	Parameter			
	BW	HW	LVW	RVW
	(g)	(mg)	(mg)	(mg)
Group				
ACF Lewis rats 11 weeks after induction of ACF	393 ± 9	2296 ± 71	1327 ± 56	528 ± 38
ACF Lewis rats 12 weeks after induction of ACF	403 ± 8	2535 ± 76	1420 ± 54	540 ± 41
ACF Lewis rats 13 weeks after induction of ACF	434 ± 12	2677 ± 55*	1512 ± 49*	675 ± 39*
ACF Lewis rats 14 weeks after induction of ACF	404 ± 9	2688 ± 54*	1542 ± 51*	686 ± 41*

The values served as basal values (100%) in evaluation of the process of cardiac atrophy in animals with HF after HT_x_ (*n*=8 in each group).

Values are means ± S.D. *P<0.05 compared with values from animals 11 weeks after induction of ACF.

**Figure 3:**
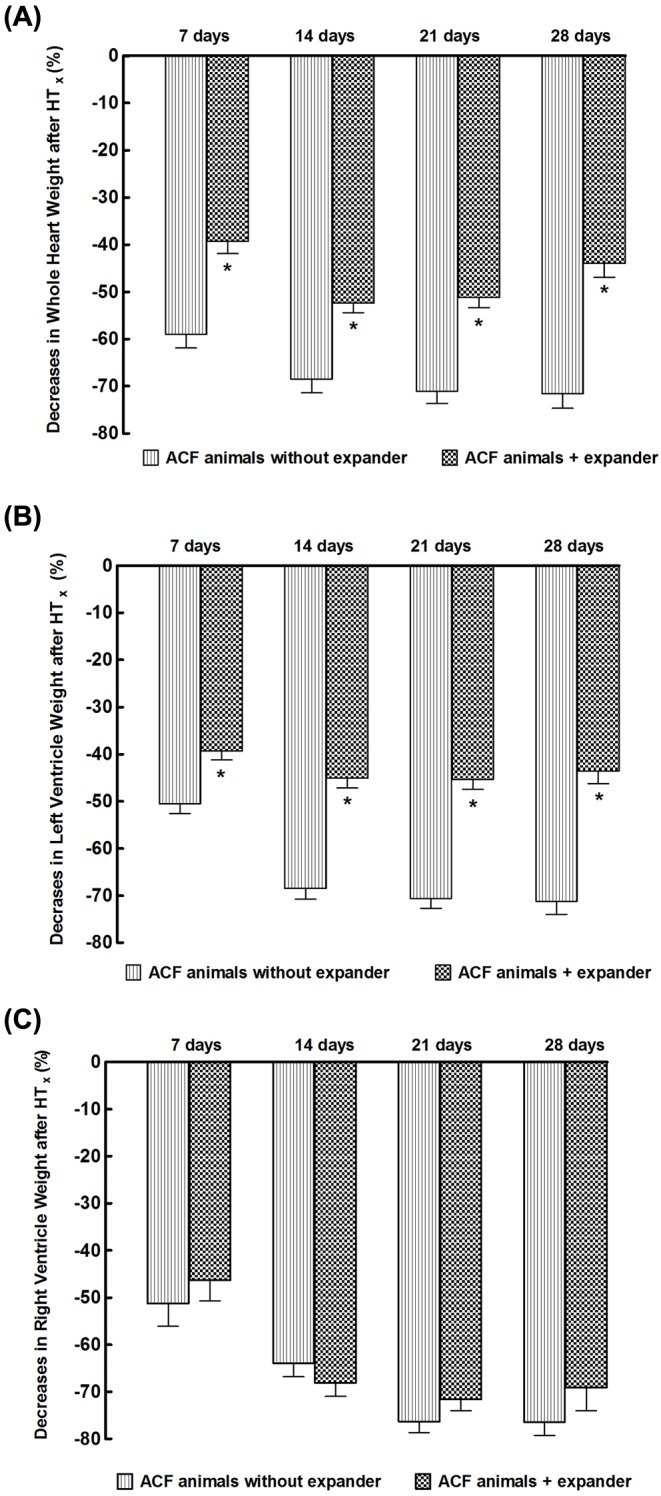
Effect implantation of the spring expander on the course of cardiac atrophy in response to mechanical heart unloading (after HT_x_) in animals with HF elicited by creation of the ACF Data are expressed as percent decreases compared with the native failing heart: (**A**) changes in whole HW, (**B**) changes in LVW, (**C**) changes in RVW. *P<0.05 compared with animals without the expander at the same time point, statistical analysis was done by Student’s *t*test, and Wilcoxon’s signed-rank test for unpaired data.

As shown in Figure 3A, 7 days’ unloading by HT_x_ in failing hearts sufficed to substantially decrease whole HW (−59 ± 3%), the decrease further progressed by day 14 (−69 ± 3%), but there was no further progress when measured on days 21 and 28 after HT_x_. The dynamics of LV and RV atrophy was quite similar as that of the whole heart (Figures 3B,C).

Figure 3A shows that implantation of the expander significantly reduced the decreases in whole HW at all time points (−39 ± 4 compared with −59 ± 4, −52 ± 3 compared with −69 ± 4, −51 ± 4 compared with –71 ± 4, and −44 ± 3 compared with −71 ± 4%, *P*<0.05 in all cases). The expander also significantly attenuated the decreases in LVW as compared with those without expander at all time points (Figure 3B). In contrast, implantation of the expander did not have any significant effect on HT_x_-induced RVW decreases in animals with ACF-induced HF (Figure 3C).

**Figure 4:**
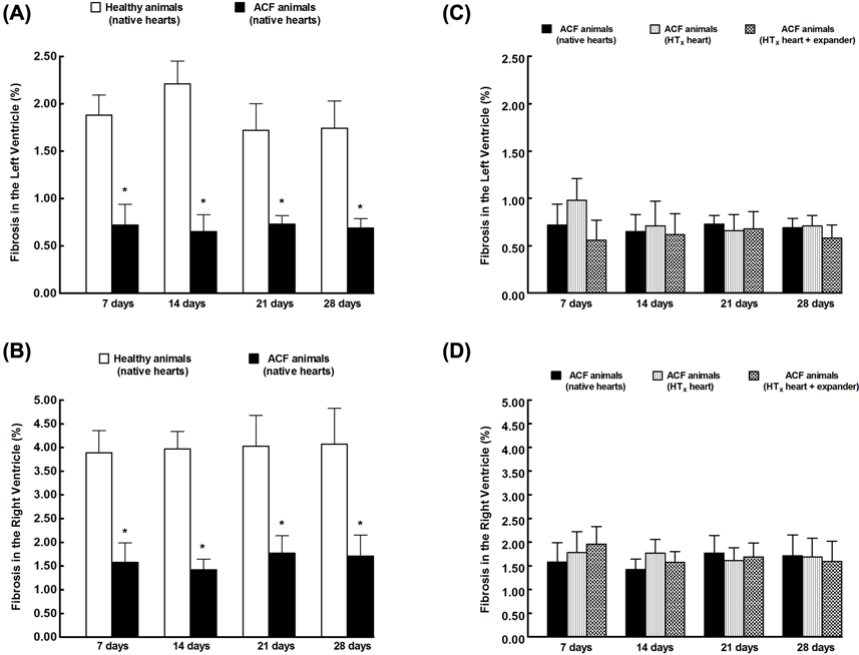
Myocardial fibrosis The index of myocardial fibrosis for the LV (**A**) and the RV (**B**) determined after the 28-day observation period in healthy animals and in animals with HF elicited by creation of the ACF. Effects of HT_x_ and implantation of the expander after HT_x_ on the degree of myocardial fibrosis in the LV (**C**) and in the RV (**D**) in the failing hearts. *P<0.05 compared with healthy animals at the same time point, statistical analysis was done by Student’s *t* test, and Wilcoxon’s signed-rank test for unpaired data.

Figure 4 summarizes the data on the index of myocardial fibrosis (%) in the LV (Figure 4A) and RV (Figure 4B) and shows that the degree of fibrosis was significantly lower in both ventricles of ACF animals as compared with healthy animals (native hearts from Lewis recipient rats) throughout the 28-day observation period. The degree of myocardial fibrosis in the LV and RV of failing hearts were not altered after HT_x_; nor did implantation of the expander after HT_x_ alter the degree of myocardial fibrosis in failing hearts (Figures 4C,D).

**Figure 5:**
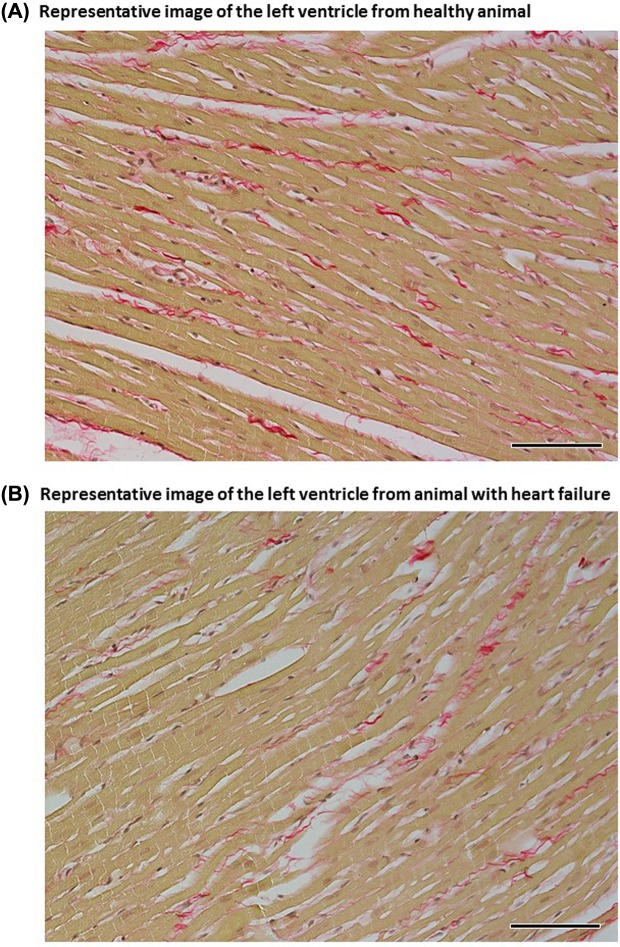
Representative images Representative images of the LV from healthy animals (**A**) and from animals with HF (**B**) elicited by creation of ACF. Sections are stained with Picrosirius Red (magnification: 200×); in these bright-field microscopy images, the collagen is red against a pale yellow background. The fibrosis in the LV of a healthy animal was estimated at 1.81% and in an ACF animal at 0.77% and these values represent a mean for eight animals in each experimental group. Scale bar in the figure is 100 µm.

**Figure 6:**
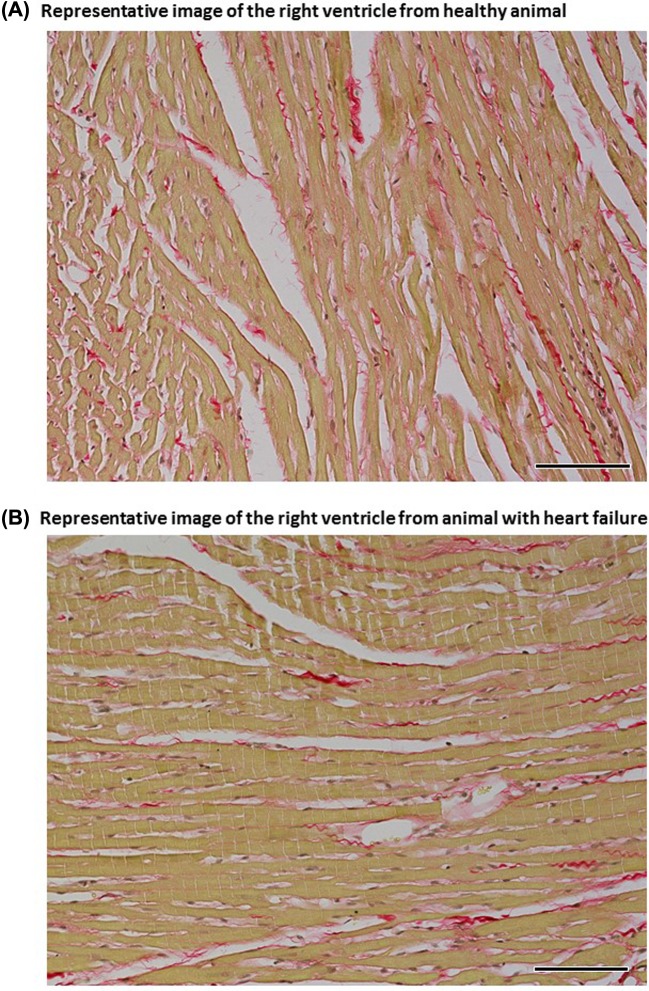
Representantive images Representative images of the RV from healthy animals (**A**) and from animals with HF (**B**) elicited by creation of the ACF. Sections are stained with Picrosirius Red (magnification: 200×); in these bright-field microscopy images the collagen is red against a pale yellow background. The fibrosis in the LV of a healthy animal was estimated at 3.92% and in an ACF animal at 1.58% and these values represent a mean for eight animals in each experimental group. Scale bar in the figure is 100 µm.

Representative images of myocardial fibrosis in the LV of healthy and failing hearts are shown in Figure 5, and and those in the RV are shown in Figure 6.

**Figure 7:**
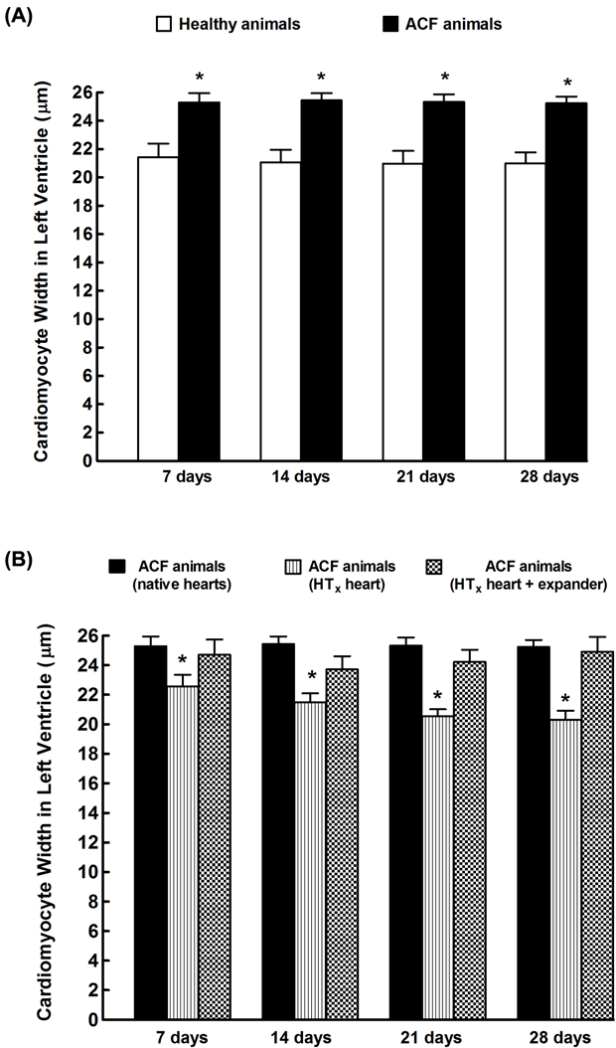
Cardiomyocyte width Cardiomyocyte width in LV from healthy animals and from animals with HF elicited by creation of the ACF (**A**) and effects of implantation of the spring expander on the LV cardiomyocyte width in mechanically unloaded hearts after HT_x_ (**B**). *P<0.05 compared with unmarked animals at the same time point; statistical analysis was done by Student’s *t*test, and Wilcoxon’s signed-rank test for unpaired data.

As shown in Figure 7A, the cardiomyocyte width values in the LV of animals with ACF-induced HF were on days 7, 14, 21, and 28 (i.e. 11, 12, 13, and 14 weeks after induction of ACF) significantly higher than those observed in healthy animals (+18 ± 3, +19 ± 3, +20 ± 3, and +19 ± 3%, respectively, *P*<0.05 in all cases).

As shown in Figure 7B, mechanical unloading of the heart after HT_x_ caused significant cardiomyocyte atrophy (a decrease in cardiomyocyte width) in the LV of the failing hearts (animals with ACF). This was already discernible on day 7 after HT_x_. It is seen that implantation of the expander prevented this width decrease.

**Figure 8:**
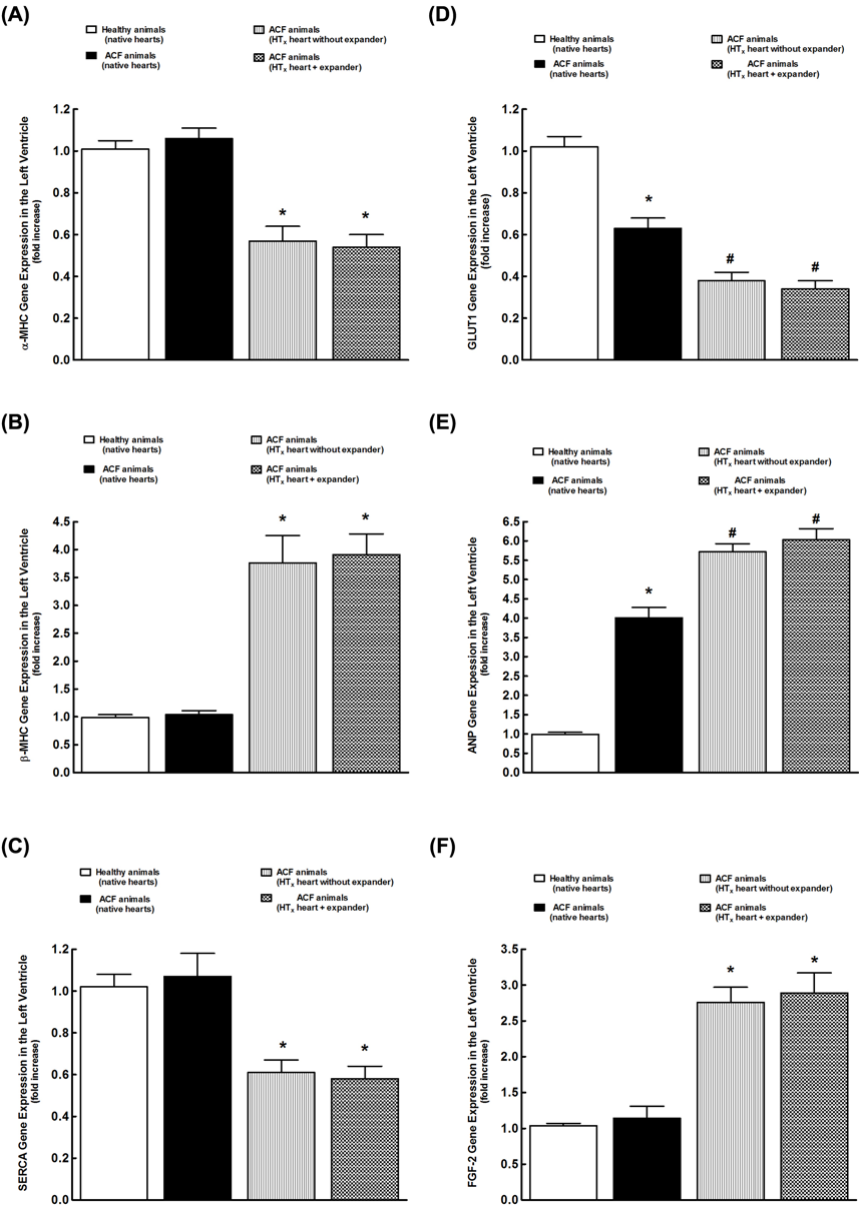
Gene expressions The LV alpha-myosin heavy chain (αMHC) (**A**), βMHC (**B**), SERCA (**C**), GLUT1 (**D**), ANP (**E**), and FGF-2 (**F**) gene expressions in the native hearts from healthy animals, and from the animals with HF elicited by creation of the ACF, and from mechanically unloaded heart after HT_x_ in ACF animals, and effects of expander implantation on these gene expressions. *P<0.05 compared with native hearts from healthy animals. ^#^*P*<0.05 compared with native hearts from ACF animals. Statistical analysis was performed by one-way ANOVA, followed by Student–Newman–Keuls test.

Figure 8A shows that there were no significant differences in the LV αMHC gene expression between healthy animals and the animals with ACF-induced HF. Mechanical unloading of the failing heart after HT_x_ resulted in significant decreases in the αMHC gene expression and implantation of the expander did not alter it.

As shown in Figure 8B, there were no significant differences in the LV βMHC gene expression between healthy animals and animals with ACF-induced HF. Mechanical unloading of the failing heart after HT_x_ caused a significant rise (more than three-fold) in the LV βMHC gene expression and it was not affected by implantation of the expander.

There were no significant differences in the LV SERCA gene expression between healthy animals and the animals with ACF-induced HF (Figure 8C). Mechanical unloading of the failing heart after HT_x_ resulted in significant decreases in the SERCA gene expression; implantation of the expander did not change this expression in the LV.

As shown in Figure 8D, LV GLUT1 gene expression was significantly lower in the hearts of the animals with ACF-induced HF than in those of healthy animals. Mechanical unloading of the failing heart further decreased LV GLUT1 gene expression in ACF animals, and implantation of the expander did not prevent this decrease. The LV gene expressions for GLUT4 and CPT I exhibited similar pattern as the GLUT1 gene expression and therefore data are not included in the figure.

Figure 8E shows that the LV ANP gene expression was four-fold higher in the animals with ACF-induced HF than in healthy animals. Mechanical unloading of the failing heart further increased the LV ANP gene expression in ACF animals and implantation of the expander did not alter it.

As shown in Figure 8F, there were no significant differences in the LV FGF-2 gene expression between healthy animals and animals with ACF-induced HF. Mechanical unloading of the failing heart after HT_x_ markedly increased the LV FGF-2 gene expression (more than 2.5-fold) and implantation of the expander did not alter it. There were no significant differences in the LV TGFβ1 gene expression between healthy animals and animals with ACF-induced HF, and mechanical unloading did not change it significantly in either group (data not shown).

## Discussion

The *first important finding* of the present study is that enhancement of isovolumic loading induced by implantation of the spring expander into the LV substantially attenuated the process of cardiac atrophy after HT_x_ in the failing heart. This was evident from higher whole HW and LVW of the transplanted heart provided with the expander than in the heart without the expander (summarized in Table 1). This effect was documented also as smaller decreases in whole HW and LVW of the transplanted heart with implanted expander compared with the decreases measured without the expander (calculated as percent decreases of transplanted heart to the control native heart from ACF animals) (summarized in Figure 3). It will also be noticed that a comparison of the degree of cardiac atrophy in the failing heart (the present study) and that observed in our recent study in the healthy (i.e. non-failing) heart [44] shows clearly that unloading-induced cardiac atrophy is distinctly more pronounced in the failing heart. These findings are critically important for at least two reasons.

First, they support our main thesis that cardiac atrophy is the most serious harmful effect of prolonged mechanical unloading which, even in the face of the sound evidence on molecular and structural ‘reverse remodeling’ after LVAD application [4,6,11,15,30], could limit successful outcomes of such treatment in terms of functional cardiac recovery and possible weaning from LVAD treatment.

Second, the results strengthen our opinion that potential anti-atrophic measures aimed at attenuation of unloading-induced cardiac atrophy should necessarily be evaluated in the failing heart, even though preparation of it for donation and, especially, heterotransplantation of such heart is technically difficult. There is no doubt that the responses of the healthy and failing hearts might be different, and the limitation related to using exclusively healthy hearts was widely admitted [11,14,16–23,28,29,35].

It is important to acknowledge that induction of isovolumic loading by implantation of the expander into the LV, as used here, was inspired by the early study by Klein et al. [23]. However, their finding that inflation in the LV of a latex balloon provided an isovolumic load and prevented heart atrophy after HT_x_ was disregarded as not applicable in the clinic: in patients with LVAD the procedure would cause obstruction of LV. Therefore, we developed the present expander which, based on preliminary studies, provides sufficient isovolumic loading and should not impair ejection function of the LV. Our hypothesis was that enhanced isovolumic loading so obtained would attenuate unloading-induced cardiac atrophy. After considering our present findings and the common knowledge that cardiac work is one of the major determinants of the size and growth of the heart [47], we propose that enhancing cardiac work due to increased isovolumic loading obtained by implantation of the expander into the LV is a reasonable approach to attenuate the development of unloading-induced cardiac atrophy, especially in the failing heart.

Nevertheless, several issues related to the morphology and function of the heart in the animals with ACF-induced HF must be addressed. This model of chronic volume overload which causes eccentric cardiac hypertrophy entails the increased length and also the width of the cardiomyocyte. The consequence is HF but, as indicated by our present and earlier results [39], unlike with pressure-induced concentric hypertrophy or ischemic cardiomyopathy induced by myocardial infarction, the process is not accompanied by myocardial fibrosis, be it in the stage of compensated cardiac hypertrophy and HF (11 weeks after induction of ACF) or in the stage of decompensated HF (21 weeks after induction of ACF) [39]. Remarkably, our present findings indicate that native hearts of ACF animals display the degree of fibrosis of the LV and RV even lower than the native hearts of healthy animals, and that HT_x_ does not alter myocardial fibrosis in the failing heart. These results contradict previous studies which showed that cardiac atrophy after mechanical unloading is associated with increased total myocardial collagen [26,27], and with the notion that the failing heart, regardless of the etiology, exhibits substantial myocardial fibrosis that contributes to increased cardiac stiffness and impaired systolic contractile and diastolic relaxation function [1,2,26,27]. The contradiction is difficult to explain, however, it should be remembered that previous experimental and clinical studies did not explore HF induced by chronic volume overload, a condition which essentially differs from HF secondary to ischemic cardiomyopathy, the most common pathomechanism of end-stage HF. Possibly, different pathophysiological background of HF could explain the different contribution of myocardial fibrosis.

Notably, the model of ACF-induced HF has recently been employed with increasing frequency because it simulates the condition of many cardiovascular patients, e.g. those with mitral insufficiency. The model has several key features of human HF: pronounced compensatory activation of neurohumoral systems, fluid retention, renal dysfunction, and gradual transition from the asymptomatic (i.e. compensated) to the decompensated phase of HF [1–3,37,38,40]. Moreover, while some clinical studies reported increased collagen content and increased myocardial stiffness after LVAD support [7,27], the results of other studies were divergent [7,48]. Overall, the effects of mechanical heart unloading on myocardial fibrosis are still a matter of debate [3–5,7] and our current findings expose some new aspects of this issue.

The***second important set of findings*** relates to the activation of the ‘*fetal cardiac gene program*’, because it is believed that returning to this gene program in the failing heart is maladaptive and plays adverse role in the development of unloading-induced cardiac atrophy [31–33]. Therefore we hypothesized that beneficial effects of increased isovolumic loading induced by implantation of the spring expander on the course of cardiac atrophy could be mediated by prevention of activation of the ‘*fetal cardiac gene program*’.

We found that there was no significant difference in the LV expression of genes related to the ‘*cardiac contractility efficiency*’ (i.e. αMHC, βMHC, and SERCA) in the native hearts of healthy and ACF animals. Mechanical unloading after HT_x_ induced profound decreases in αMHC and SERCA gene expression and marked increases in *βMHC* gene expression in the LV, which is in accordance with previous studies [7,27,31–33], but implantation of the spring expander did not prevent these changes. On the contrary, the expression of genes regulating ‘*cardiac substrate uptake and substrate oxidation*’ in the LV [46] (i.e. GLUT1, GLUT4, and CPT I) was substantially lower in the failing as compared with the healthy hearts, and the mechanical unloading further decreased their gene expression in the LV; implantation of the spring expander did not attenuate these decreases. In addition, the LV gene expression for ANP, a recognized marker for the degree of pathological remodeling [1,3], was markedly increased in failing hearts and was further augmented by the mechanical unloading. Again implantation of the expander did not prevent or attenuate these changes. Taken together, our present results are in agreement with previous studies showing that mechanical unloading induces activation of the ‘*fetal gene cardiac program*’ [31–33] and it was suggested that this return is detrimental and a hallmark of progressive deterioration of cardiac functions [31–33]. However, the entirely novel finding here is that even though the enhancement of isovolumic loading induced by implantation of the expander into the LV did attenuate the process of cardiac atrophy after HT_x_ in the failing heart, it did not prevent or even reduce the degree of activation of the ‘*fetal gene cardiac program*’. Thus, these findings suggest that, unlike in our original hypothesis, the beneficial effects of the expander implantation on the course of unloading-induced cardiac atrophy are not related to the prevention of activation of the ‘*fetal gene program*’. However, one should be aware that superordinate regulators of global gene networks, miRNAs, acting mainly at the translation level, have been identified and El-Armouche et al. [49] showed that opposite changes in cardiac work induced either by mechanical overloading or unloading are associated with different activity of specific sets of miRNAs rather than with the degree of activation of the ‘*fetal gene cardiac program*’. Therefore, any future studies should also address the possible role of miRNAs in the mediation of beneficial effects of the expander on the course of unloading-induced cardiac atrophy.

**Limitations of the study and the prerequisites for potential translation to clinical practice**

Apart from the above discussed reservations, two limitations of the present study should be defined before clinical application of the expander could be considered.

**A major limitation** is the lack of assessment of cardiac function after HT_x_: this could help explain why long-term implantation of the expander does not impair ejection function of the LV, which actually was observed with inflated latex balloon as employed by Klein et al. [23]. It has to be emphasized that our studies, admittedly of preliminary nature, were limited to evaluation of cardiac function after acute implantation of the expander, without exploring the effects of long-term implantation. Complex assessment of cardiac function would provide important information on the mechanical properties of chronically unloaded heart and effects of long-term implantation of the expander. However, in this context original studies by Korecký and Rakušan [50] and other groups should be recalled. Using an array of imaging and functional assessment techniques these workers showed that despite considerable atrophy, the intrinsic contractile properties of the myocardium apparently remained normal [4,14,15,17,19,20,25,30,35,51]. In addition, quantitative histologic studies showed that cardiac atrophy after HT_x_ is due to the loss of volume of existing myocytes without any significant change in their number [15,29,35,42,50]; therefore cardiac atrophy is considered the most detrimental effect of prolonged heart unloading [4,9,11,14,15,22,30,51]. Since a close relationship between the decrease in the myocyte volume and of the heart mass has been clearly shown [15,29,47,50], it is commonly accepted that ‘unsophisticated’ markers such as whole heart, LV and RV weights, when compared with the respective values in the recipient, are reliable indices of the function of the heart exposed to long-term unloading after HT_x_ [11,30,51]. Nevertheless, it is not known if and how implantation of the expander would alter cardiac mechanical and metabolic functions. Therefore, more studies employing various imaging and functional assessment techniques would be needed (including evaluation of the length–tension relationship of the isolated papillary muscle, and transabdominal echocardiography followed by positron emission tomography) before considering implantation of the expander in clinical practice. The present study provides a necessary basis for such studies.

**Another limitation** relates to the models of HT_x_ and HF. Attention is drawn to harmful effects of exposure to ischemia/reperfusion and to possible beneficial effects of cardiac denervation: obviously, in the clinical setting the unloaded heart remains innervated [30,51]. Nevertheless, these limitations of the HT_x_ model might not critically influence the experimental results. More important is that the failing heart is transplanted into a healthy recipient. Thus, the organ derived from animals with advanced HF with abnormal activation of the circulatory hormonal systems [1,3] is abruptly placed in the normal neurohormonal environment. Dissimilarly, in patients with implanted LVAD the abnormal neurohormonal milieu is maintained [1,3,6]. Possibly, such differences in neurohormonal environment might alter the responses of the transplanted heart to expander implantation. Remarkably, clinical studies have shown that the ‘reverse remodeling’ and functional recovery of the LV function in patients after LVAD implantation were enhanced by intense pharmacotherapies aimed at normalization of the neurohormonal milieu in patients with implanted LVAD [6,52]. Moreover, it is remembered that HF is a clinical syndrome which originates from diverse cardiovascular diseases [1–3] and our present results might be valid only for the failing heart characteristic for the ACF-induced HF model. Therefore, future studies are needed with HF-affected recipient animals: a difficult task that has not yet been successful. Moreover, another model of HF for the donor as well as recipient will have to be employed (e.g. HF induced by pressure overload or myocardial infarction). These are the prerequisites for considering implantation of the expander in patients with LVAD-induced cardiac atrophy.

On the whole, we have to acknowledge that our present data do not clarify the exact mechanism(s) underlying the beneficial effects of the expander on the course of unloading-induced cardiac atrophy. Nevertheless, while acknowledging the reservations discussed above we have to consider the established knowledge that the cardiac work is a major determinant of the size and growth of the heart [47]. Therefore we propose that enhancing this work by increasing isovolumic loading obtained by implantation of the expander might be a reasonable approach to attenuate the unloading-induced cardiac atrophy.

## Clinical perspectives

- The development of LV atrophy is the most common complication of the prolonged LVAD support in patients with advanced HF. A variety of treatment approaches were used to attenuate the progression of atrophy, however, none of these attempts brought satisfactory results.
- Our present study shows that induction of isovolumic loading by implantation of the three-branch spring expander into the LV attenuates the process of unloading-induced cardiac atrophy in rats with advanced HF.
- Our findings provide experimental basis for a new therapeutic approach toward minimizing the detrimental effects of the LVAD application in patients with HF.

## Abbreviations

ACF: aorto-caval fistula
ANP: atrial natriuretic peptide
CPT I: carnitine palmitoyltransferase I
FGF-2: fibroblast growth factor type 2
GLUT1: glucose transporter type 1
GLUT4: glucose transporter type 4
HF: heart failure
HT_x_: heterotopic heart transplantation
HW: heart weight
LV: left ventricle
LVAD: LV assist device
LVW: LV weight
MHC: myosin heavy chain
RV: right ventricle
RVW: RV weight
SERCA: sarcoendoplasmatic Ca^2+^-ATPase pump
TGFβ1: transforming growth factor β1
